# Recommendations on clinical trial design for treatment of Mucopolysaccharidosis Type III

**DOI:** 10.1186/s13023-017-0675-4

**Published:** 2017-06-26

**Authors:** Arunabha Ghosh, Elsa Shapiro, Stewart Rust, Kathleen Delaney, Samantha Parker, Adam J Shaywitz, Adelaida Morte, Gillian Bubb, Maureen Cleary, Tien Bo, Christine Lavery, Brian W Bigger, Simon A Jones

**Affiliations:** 1 Willink Biochemical Genetics Unit, Manchester Centre For Genomic Medicine, Manchester Academic Health Science Centre, Central Manchester University Hospitals NHS Foundation Trust, Manchester, UK; 2 School of Biological Sciences, Faculty of Biology, Medicine and Health, University of Manchester, Manchester, UK; 3 Shapiro & Delaney LLC, Mendota Heights, MN USA; 4 Paediatrics and Neurology, University of Minnesota, Minneapolis, MN USA; 5 Paediatric Psychosocial Service, Royal Manchester Children’s Hospital, Central Manchester University Hospitals NHS Foundation Trust, Manchester, UK; 6 Lysogene, Paris, France; 7 BioMarin Pharmaceutical Inc., Novato, CA USA; 8 Esteve, Barcelona, Spain; 9 Alexion Pharmaceutical, New Haven, CT USA; 10 Great Ormond Street Hospital, London, UK; 11 Shire, Lexington, MA USA; 12 MPS Society, Amersham, UK; 13 Stem Cell & Neurotherapies, Division of Cell Matrix Biology and Regenerative Medicine, Faculty of Biology, Medicine and Health, University of Manchester, Manchester, UK

**Keywords:** Lysosomal storage disorders, mucopolysaccharidoses, Mucopolysaccharidosis Type III, Sanfilippo syndrome, Clinical trials, Natural history, Quality of life, Cognitive assessment, Behaviour

## Abstract

**Background:**

Mucopolysaccharidosis type III is a progressive, neurodegenerative lysosomal storage disorder for which there is currently no effective therapy. Though numerous potential therapies are in development, there are several challenges to conducting clinical research in this area. We seek to make recommendations on the approach to clinical research in MPS III, including the selection of outcome measures and trial endpoints, in order to improve the quality and impact of research in this area.

**Results:**

An international workshop involving academic researchers, clinical experts and industry groups was held in June 2015, with presentations and discussions on disease pathophysiology, biomarkers, potential therapies and clinical outcome measures. A set of recommendations was subsequently prepared by a working group and reviewed by all delegates. We present a series of 11 recommendations regarding the conduct of clinical research, outcome measures and management of natural history data in Mucopolysaccharidosis type III.

**Conclusions:**

Improving the quality of clinical research in Mucopolysaccharidosis type III will require an open, collaborative and systematic approach between academic researchers, clinicians and industry. Natural history data should be published as soon as possible and ideally collated in a central repository. There should be agreement on outcome measures and instruments for evaluation of clinical outcomes to maximise the effectiveness of current and future clinical research.

## Introduction

Mucopolysaccharidosis III (MPS III), or Sanfilippo syndrome, is a progressive, neurodegenerative lysosomal storage disorder caused by an inherited defect in the degradation of heparan sulfate (HS). MPS III is the most common mucopolysaccharidosis with an incidence of 0.27–1.89 per 100,000 live births [1–11]. Each of the four variants of the disorder (MPS IIIA, B, C, D) is associated with the deficiency of a specific enzyme involved in HS degradation (N-sulfoglucosamine sulfohydrolase (SGSH), EC 3.10.1.1, MPS IIIA; N-acetyl-α-glucosaminidase (NAGLU), EC 3.2.1.50, MPS IIIB; heparan-α-glucosaminide N-acetyltransferase (HGSNAT), EC 2.3.1.78, MPS IIIC; N-acetylglucosamine-6-sulfatase (GNS), EC 3.1.6.14, MPS IIID). The most common subtype differs between geographical areas but is either MPS IIIA or IIIB [5, 12]. MPS IIIC is less common and MPS IIID is the rarest [13–15].

The principal biochemical abnormality is the accumulation of lysosomal HS, but secondary storage products, particularly GM2 and GM3 gangliosides, are also thought to play a role in central nervous system (CNS) pathology [16, 17]. CNS pathophysiology likely involves a multiplicity of mechanisms including neuroinflammation, defective autophagy and mitochondrial dysfunction [18–22].

Neurological disturbance dominates the clinical picture, typically described as progressing in three phases [23]. In the first phase, beginning at 1–4 years of age, there may only be delayed development, particularly of speech and language. The second phase begins at 3–5 years and is characterised by marked behavioural disturbance with aggression, hyperactivity and sleep disturbance, and the beginning of progressive cognitive decline [24–29]. Finally, in the third phase, beginning from around 10 years onward, there is progressive loss of motor function, eventually resulting in complete loss of ability to walk, swallowing and feeding difficulties often requiring gastrostomy feeding, and there may be seizures [24, 28, 29]. Death usually occurs in the second decade, except in attenuated patients.

The four subtypes are clinically indistinguishable, though progression of disease is possibly slower in MPS IIIC [27–29]. However, there is a great degree of variability both within and between subtypes [25, 27]. Very attenuated phenotypes have been reported, usually in association with specific mutations [30–34]. Specific mutations associated with severe or attenuated phenotypes are well recognised in MPS IIIA [30] but not MPS IIIB.

No approved disease-modifying treatments exist. Standard enzyme replacement therapies (ERTs) do not cross the blood-brain barrier, limiting their applicability in this predominantly CNS disease. Haematopoietic stem cell transplantation (HSCT) is not effective in MPS III [35, 36], even when performed before the onset of neurological manifestations [37]. Numerous treatment approaches are under investigation in animal models and humans, including CNS-directed ERT [38–41]; substrate reduction therapy (SRT) [42, 43] and gene therapy [44–53], with several progressing to clinical trial [38, 51, 54–56] (Table 1).

**Table 1:** Summary of published and ongoing interventional studies in humans and endpoints selected

Reference / clinical trial registry identifier and sponsor	Subtype (n)	Phase	Intervention	Design	Comparator group	Primary endpoint	Additional endpoints
Guffon et al. (2011) [104] Hospices Civiles de Lyon (IMP supplied by Actelion)	MPS III A,B,C,D (25)	II/III	Oral miglustat	Randomised, double blinded, placebo controlled efficacy study	Placebo	Behavioural disturbance using VABS-II	Sleep disturbance (questionnaire) Hyperactivity (Conners scale) Developmental age (Borel Maisonny Petit test) Brain MRI GM2 ganglioside and Miglustat concentrations in CSF
Tardieu et al. (2014) [51] Lysogene	MPS IIIA (4)	I/II	Gene therapy: intracerebral AAV rh.10-SGSH-IRES-SUMF1 vector	Open label tolerance/safety study over 1 year follow up	None	Tolerance and safety of IMP and procedure: - frequency of adverse events - formal clinical and neurological examinations - standard haematological and biochemical parameters - immunovirology - CSF protein, glucose, cell count and IFNα titer - routine urinalysis	Changes in brain atrophy evaluated by MRI Neuropsychological meaures: PEP-3, VABS-II, TBAQ, Brunet-Lezine revised test Viral titres in urine Anti-AAV rh.10 antibodies CSF stored for future biomarker analysis
Jones et al. (2016) [38] Shire	MPS IIIA (12)	I/II	Intrathecal heparan-N-sulfatase (rhHNS)	Open label, dose escalation safety study over 6 month follow up	None	Safety and tolerability of intrathecal administration of rhHNS: - adverse events - CSF cell counts and chemistries - echocardiography - anti-rhHNS antibodies in CSF and serum	Neurocognitive assessments (Eligibility determined by VABS-II, enrolled patients tested using BSID-III or KABC-II depending on age or developmental age from VABS-II) Cortical grey matter volume by MRI Serum PK CSF HS (by LC-MS/MS) uGAG by DMB assay
NCT01299727 (ClinicalTrials.gov) [105] Shire	MPS IIIA (12)	I/II	Intrathecal heparan-N-sulfatase (rhHNS)	Open label, long term safety and tolerability study	None	Adverse events Clinical laboratory including liver function tests, haematology, and urinalysis Electrocardiography CSF chemistries, cell counts and inflammatory markers Anti-rhHNS antibodies in CSF and serum	Standardized neurocognitive and behavioural assessments (Sanfilippo-specific behavioural rating scales, gross and fine motor assessments, functional adaptive rating scales, quality of life questionnaires, and Children’s Sleep Habits Rating Scale) rhHNS concentration in CSF and serum Inflammatory cytokines in serum and CSF Safety and potential surrogate efficacy biomarkers in CSF, urine, and serum HS and HS derivatives in urine, plasma and serum Brain (MRI) and auditory brainstem response
NCT02060526 (ClinicalTrials.gov) [98] Shire	MPS IIIA (21, 7 in each arm)	II	Intrathecal heparan-N-sulfatase (rhHNS)	Randomised, controlled, open label safety and efficacy study in patients with early stage MPS IIIA	3 arms: Placebo 2-weekly rhHNS 4-weekly rhHNS	Maximum decline in DQ of 10 points over 48 weeks, assessed by neurocognitive testing	Adverse events Anti-rhHNS antibodies in serum Adaptive behavioural function assessed by neurocognitive testing Total cortical grey matter volume by MRI GAG concentrations in CSF and urine rhHNS concentrations in CSF and serum
2013–001479-18 (EudraCT) [56] CMFT	MPS III A,B,C (21)	III	High dose oral genistein aglycone	Randomised, double blinded placebo controlled efficacy study with partial crossover design	Placebo controlled for 12 months followed by open label treatment phase	Change in CSF HS at 52 weeks	CSF HS at 104 weeks uGAG by DMB assay HS in urine and plasma Cognitive function using BSID-III and adaptive behaviour using VABS-II, MPS symptom checklist Actigraphy
NCT02324049 (ClinicalTrials.gov) [55] Alexion	MPS IIIB (9)	I/II	Intravenous recombinant human alpha-N-acetylglucosaminidase (rhNAGLU)	Open label safety/efficacy study	None	Safety and tolerability - physical examinations - electrocardiography - clinical laboratory tests - concomitant medications - anti-drug antibodies	PK/PD effects Neurocognitive and developmental function Brain structure volumetric assessment Indices of microstructural integrity
NCT02618512 (ClinicalTrials.gov) [106] Alexion	MPS IIIB (5)	I/II	Intravenous recombinant human alpha-N-acetylglucosaminidase (rhNAGLU)	Open label safety/efficacy study	None	Safety and tolerability - physical examinations - electrocardiography - clinical laboratory tests - concomitant medications - anti-drug antibodies	PK/PD effects Neurocognitive and developmental function Brain structure volumetric assessment Indices of microstructural and blood-brain barrier integrity
NCT02754076 ClinicalTrials.gov [107] Biomarin	MPS IIIB (33)	I/II	Intracerebroventricular infusion of recombinant human alpha-N-acetylglucosaminidase and truncated human insulin-like growth factor 2 (rhNAGLU-IGF2)	Open label dose escalation safety and efficacy study	None	Safety evaluation - treatment related adverse events or abnormal clinical laboratory tests Rate of change of DQ score on treatment vs. rate of change of DQ score prior to treatment assessed at study end (124 weeks)	Concentrations of IMP in CSF and plasma Anti-drug antibodies in CSF and serum GAG concentrations in CSF, plasma and urine Brain structure by MRI
NCT02716246 ClinicalTrials.gov [54] Kevin Flanigan / Abeona Therapeutics	MPS IIIA (9)	I/II	Gene therapy: intravenous scAAV9.U1a.hSGSH vector	Open label, dose escalation safety and efficacy study	None	Development of unacceptable toxicity	CSF and leukocyte SGSH enzyme activity Liver and spleen volumes by MRI Adaptive functioning by VABS-II Cognitive function by Leiter International Performance Scale and Mullen Scales of Early Learning uGAG and urine HS

*Key: AAV* adeno-associated virus, *IMP* investigational medical product, *PEP-3* psychoeducational profile, 3rd edition, *VABS-III* Vineland Adaptive Behaviour Scale, 2nd edition, *TBAQ* Toddler Behaviour Assessment Questionnaire, *IFNα* interferon alpha, *KABC-II* Kaufman Assessment Battery for Children, Second Edition, *BSID-III* Bayley Scales of Infant and Toddler Development, Third Edition, *uGAG* urinary glycosaminoglycan, *DMB* dimethylmethylene blue, *CSF* cerebrospinal fluid, *LC-MS/MS* liquid chromatography-tandem mass spectrometry, *EudraCT* European Union Drug Regulating Authorities Clinical Trials, *CMFT* Central Manchester University Hospitals NHS Foundation Trust, *PK* pharmacokinetics, *DQ* Developmental Quotient, *PD* pharmacodynamics

Given the phenotypic heterogeneity, progressive nature of disease and small numbers of patients, understanding the natural history of the disease is essential for informing clinical trial design and selection of appropriate outcome measures. Natural history studies provide comparison data and potentially form historical control groups for interventional studies. Though several natural history studies have been undertaken, they differ in design and selection of parameters measured, and not all are published (Table 2). Earlier studies collected retrospective data, often based on parent/caregiver recollection and not methodologically robust enough to form comparator groups. To date, only two published studies have prospectively collected individual, longitudinal natural history data [57, 58]. The majority of published detailed natural history data is for MPS IIIA.

**Table 2:** Summary of design and parameters measured in natural history studies in MPS III

Reference	Geographical region(s)	Subtype(s) (n)	Design	Parameters measured
Meyer et al. (2007) [29]	Germany	MPS IIIA (71) MPS IIIB (14) MPS IIIC (4)	Retrospectively collected data based on family interviews using a questionnaire and FPSS	Questionnaire: Family history, onset of symptoms, diagnostic tests, physical symptoms, developmental delay, behavioural problems, sleep disturbance, neurological symptoms, physical development/puberty, therapies and medications, social care, developmental regression, death FPSS: motor function, speech abilities, cognitive function; assessed retrospectively over course of disease at 3–6 month intervals
Ruijter et al. (2008) [28]	Netherlands	MPS IIIC (29)	Retrospectively collected data based on paediatrician and geneticist interview with family using a questionnaire, or case note review for deceased patients	Questionnaire: pregnancy, first clinical signs and symptoms, mental and motor milestones, behavioural and sleep problems, medical history DNA sequencing HGSNAT activity in fibroblasts
Malm et al. (2010) [2]	Sweden	MPS IIIA (15) MPS IIIB (1) MPS IIIC (5)	Retrospective case notes review with completion of missing data by interview with families	Motor function and loss of ability, speech development and loss of ability, mental development using IQ measures from locally applied neuropsychological tests (not specified), clinical symptoms and somatic features including behavioural problems, sleep, epilepsy, umbilical or inguinal hernia, orthopaedic problems Enzyme assay in leukocytes or fibroblasts
Valstar et al. (2010) [64]	Netherlands	MPS IIIB (52) (majority with attenuated phenotype)	Retrospectively collected data based on clinician interview with family using a questionnaire, or case note review for deceased patients	Questionnaire: pregnancy and delivery, first clinical signs and symptoms, mental and motor milestones, behavioural problems, sleeping problems, medical history Clinical examination DNA sequencing
Valstar et al. (2010) [30]	Netherlands	MPS IIIA (110)	Retrospectively collected data based on clinician interview with family using a questionnaire, or case note review for deceased patients	Questionnaire: pregnancy, first clinical signs and symptoms, mental and motor milestones, behavioural problems, sleeping problems, medical history FPSS as used in Meyer et al. (2007) Clinical examination DNA sequencing
Heron et al. (2011) [5]	France, UK, Greece	France - MPS IIIA (76) - MPS IIIB (16) - MPS IIIC (13) - MPS IIID (6) UK - MPS IIIA (89) - MPS IIIB (22) - MPS IIIC (7) - MPS IIID (2) Greece - MPS IIIB (16) - MPS IIIC (3)	France, Greece: retrospectively collected data from participating physicians without direct contact with families using pre-established form UK: retrospectively collected data from telephone interview with families conducted by UK MPS Society using pre-established form	Pre-established form: Presence of specified clinical features at diagnosis and occurrence during follow up, e.g. early language, loss of walking, cognitive delay, abnormal behaviours, school level Enzyme activity in leukocytes or fibroblasts DNA sequencing
Grant et al. (2013) [81]	UK	Parents (23) of children with MPS III (19) Parents (23) of children with ID (20)	Child behaviour and parental psychological functioning. Questionnaire packs sent out to families and completed at a single time point.	Behaviour: LDCMS, ECBI Parental psychological functioning: Resilience Scale for Adults, Multidimensional Scale of Perceived Social Support, coping techniques (Brief COPE questionnaire), Paediatric Inventory for Parents, parental anxiety and depression (General Health Questionnaire, GHQ-12)
Delgadillo et al. (2013) [24]	Spain	MPS IIIA (34) MPS IIIB (11) MPS IIIC (10)	Retrospectively collected data using a questionnaire answered by physicians and parents	Questionnaire: early psychomotor development, age at diagnosis, first clinical symptoms, somatic features, speech, behavioural and sleep disturbance, evolution of neurodegenerative symptoms, age at regression of acquired skills and loss of functional abilities, feeding, cognitive failure through the disease, orthopaedic complications, death Details of previously conducted enzyme assays and mutation analysis
Mahon et al. (2014) [76]	UK	MPS IIIA (4) MPS IIIB (4)	Prospective study over 7 days of eight children with MPS III and eight age-matched typically developing controls	Actigraphy Salivary melatonin concentrations Sleep questionnaire and daily sleep diary
Cross et al. 2014 [108]	UK	MPS III (20) ID (24)	Assessment of behaviour and adaptive skills. Questionnaire packs sent out to families and completed at a single time point.	Behaviour: LDCMS, ECBI, Aberrant Behaviour Checklist, SBRS Adaptive skills: VABS-II
Buhrman et al. (2014) [60]	USA	MPS IIIA (46)	Retrospectively collected data using a standardised protocol of assessments conducted at a single visit by an MDT (neurodevelopmental pediatricians, speech and language pathologists, developmental specialists, psychologists, audiologists, physical therapists)	Age at diagnosis and initial symptoms, Audiology assessments Cognitive function, adaptive behaviour, expressive and receptive language, motor development (neuropsychological instruments not specified) Behavioural symptoms Somatic symptoms Growth parameters (height, weight, BMI, head circumference) Survival
Mumford et al. (2015) [77]	UK	MPS IIIA (4) MPS IIIB (4)	Prospective study of children with MPS III and age-matched typically developing controls over 7–10 days	Actigraphy
Shapiro et al. (2016) [58]	USA	MPS IIIA (25)	Longitudinal data collected prospectively over a 2 year follow up period with evaluations at baseline, 6 months, 12 months and 2 years	Cognitive assessments (KABC-II or BSID-III), reported as age equivalent scores and DQs Adaptive behaviour (VABS-II), age equivalent scores and DQs Disability (FPSS four-point scoring system) Sleep Habits Questionnaire Volumetric MRI of brain (cortical grey matter volume, white matter volume, ventricular volume) Volumetric MRI liver and spleen Total uGAG by DMB assay CSF biomarkers (HS by LC-MS/MS; T-tau, p-tau by immunoassay)
Truxal et al. (2016) [57]	USA	MPS IIIA (15) MPS IIIB (10)	Longitudinal data collected prospectively over a 1 year follow up period with evaluations at baseline, 6 months and 12 months	Medical history, physical examinations conducted by a physician trained in neurology or genetics Cognitive assessments (Leiter-3 or Leiter-R, reported as age-normed standard scores; later amended to Mullen Scales of Early Learning reported as age equivalent scores) Adaptive behaviour (Achenbach Child Behaviour Checklist, reported as age and gender-normed T-scores; or Adaptive Behaviour System, but removed in favour of VABS-II, reported as age-normed composite standard scores) Pediatric Evaluation of Disability Inventory (PEDI), timed motor function tests (timed 10 min walk, 6MWT, timed ascent/descent of 4 stairs) MRI volumetric measurements of liver and spleen MR/MR spectroscopy of brain (no longitudinal volumetric studies of brain regions) Echocardiography SGSH or NAGLU enzyme activity in CSF SGSH enzyme activity in leukocytes or NAGLU enzyme activity in plasma uGAG by DMB assay Complete blood count, coagulation studies, liver enzyme tests in blood CSF glucose, protein and cell count Routine urinalysis

*Key: FPSS* four point scoring system, *BMI* body mass index, *KABC-II* Kaufman Assessment Battery for Children, Second Edition, *BSID-III* Bayley Scales of Infant and Toddler Development, Third Edition, *DQ* developmental quotient, *uGAG* urinary glycosaminoglycan, *DMB* dimethylmethylene blue, *CSF* cerebrospinal fluid, *LC-MS/MS* liquid chromatography-tandem mass spectrometry, *VABS-II* Vineland Adaptive Behaviour Scale, Second Edition, *6MWT* 6 min walk test, *ID* intellectual disability, *LDCMS* Learning Disability Case Mix Scale, *ECBI* Eyberg Child Behaviour Inventory, *SBRS* Sanfilippo Behaviour Rating Scale

Clinical trials are a necessity in MPS III given the lack of effective therapies. Phenotypic heterogeneity, small patient numbers, the multidisciplinary management of the disease, the multiplicity of potential approaches to treatment and the difficulty of assessing the effects of treatments targeted to the CNS all present challenges to conducting research in this area. There is therefore a need for consensus on how best to move forward with clinical research, collate natural history data, and select robust and clinically meaningful outcome measures for clinical trials.

## Methods

An international workshop on MPS III clinical research was held in June 2015 to develop collaborations between academic research, clinical experts and industry groups, and work towards recommendations on conducting clinical research in MPS III. This was funded by the UK MPS Society and received no commercial grants. Invited delegates were members of all academic, clinical and industry groups known to be involved with a current or upcoming clinical research programme in MPS III, as well as representatives from the UK MPS Society. Experts included paediatricians for metabolic disorders, neuropsychologists and academic researchers with an acknowledged expertise in MPS III. The workshop programme and a full delegate list are included in supplementary information. Presentations included updates on research by academic, clinical and industry groups, and were followed by active discussion sessions with a view to developing recommendations for clinical trials. All groups of delegates participated in discussions. Recommendations were then formulated by a working group established at the workshop and fed back to all delegates for comment. Disagreements were resolved by discussion. Final recommendations represent a combination of published data and the experience of experts in MPS III. Further experience and an increasing evidence base may allow for consensus to be developed for neurocognitive endpoints using a formal Delphi method and a consensus conference with this goal has been organised (Elsa Shapiro, personal communication).

## Recommendations

1. Due to the universal presence of CNS manifestations in MPS III patients and their overall greater impact on morbidity and mortality, the effect on neurological disease should be the focus of outcome measures and trial endpoints in current MPS III research, though the effect of potential therapies on somatic manifestations should be taken into account.

CNS manifestations predominate in MPS III, particularly intellectual disability, progressive loss of acquired skills, behavioural disturbance and sleep disturbance. The reduced lifespan in severely affected patients is likely to be related to progressive neurological deterioration, with most deaths being due to bronchopneumonia (Christine Lavery, personal communication).

However, the involvement of multiple organ systems in MPS III is well recognised [59]. Most patients have somatic manifestations [5, 60], the most frequent being coarse facial features and hepatomegaly. Recurrent ENT infections and diarrhoea are common symptoms and a significant number of patients undergo adenoidectomy or tonsillectomy [60]. Umbilical or inguinal hernias are frequently reported [25, 29, 30]. Multiple skeletal manifestations have been described, particularly hip dysplasia and osteonecrosis of the femoral head [23, 61, 62].

The relevance of somatic manifestations may become increasingly important with the development of CNS-directed therapies. Manifestations such as cardiac valvular abnormalities, hearing loss and retinopathy [60, 63, 64] may be a significant contribution to morbidity in attenuated patients living for longer with slowly progressive disease. Somatic features such as skeletal disease and sleep disordered breathing may also be potential confounders of CNS outcomes such as neurodevelopmental assessments, and should be accounted for [65].

2. Both cognitive and behavioural manifestations should be considered in the development of clinical trial endpoints.

Cognitive and behavioural manifestations are both well recognised in MPS III, and, except for impaired acquisition of language, appear to develop within a similar timeframe. The onset of both delayed cognitive development and abnormal behaviours occurs between approximately 3–4 years of age on average, though there is a great degree of variability [2, 5, 24, 27–30, 58, 60, 64]. In MPS IIIA, adaptive behaviour remained intact for longer than cognitive function in one study [60], but in a more recent study, children continued to acquire adaptive behaviour skills for longer than cognitive skills, though both declined after around 4 years [58].

Though cognitive and behavioural manifestations are related, they are different and may impact differently on quality of life [66]. Cognitive impairment may also confound assessments of behaviour [67] and vice versa. Assessments of both cognitive function and behaviour should therefore be considered important outcome measures. However, in clinical trials where sample sizes are small, phenotypic variability may limit the applicability of cognitive and behavioural assessments as primary endpoints. Neurological symptoms such as seizures, ataxia and dystonia appear late in the disease course and therefore are of limited use as outcome measures, particularly where the goal is to assess response to early therapy.

3. Cognitive and behavioural manifestations are less evident below the age of 2 years. For these patients, cognitive and behavioural assessments should be used as long term assessments over a period of years following identification.

Cognitive manifestations are unlikely to become apparent before 2 years of age, other than a subtle slowing in the development of cognitive and language skills or failure to acquire language at all [24, 30]. Children may continue to acquire skills beyond 2 years [57, 58], and in patients with slowly progressive disease, manifestations may not become apparent for several years.

Where interventional trials involve children under 2 years of age, cognitive and behavioural assessments should therefore be used as long term outcome measures over a period of years. Development of shorter-term measures of treatment effects in this age group will be of value, though this needs to be coupled with a better understanding of genotype-phenotype correlations to predict slowly or rapidly progressive disease. One approach may be to determine whether children continue to acquire skills beyond an expected ceiling of development. In MPS IIIA, the ceiling of cognitive development is reported as 36 months in children with rapidly progressive phenotype [58]. In contrast, there is little published natural history data on cognitive development in MPS IIIB children under 6 years.

4. Cognitive assessment should be used as a long-term endpoint, using the Bayley Scales of Infant and Toddler Development (3rd edition) and/or the Kaufman Assessment Battery for Children (2nd edition). The Vineland Adaptive Behaviour Scale is a useful additional measure, but cannot be the only developmental endpoint. We advocate the use of age equivalent scores to assess the relative rate of development.

Delayed development followed by progressive loss of skills is characteristic of MPS III and developmental endpoints are therefore important for evaluating the efficacy of potential treatments.

There are several challenges to administering cognitive assessments in this patient population. Motor ability, language regression, hearing or visual impairment, and emotional or behavioural factors may influence the child’s performance, as may environmental factors such as recent medical procedures, general anaesthesia or sedation, or fatigue [27, 65, 67–69]. Testers must be familiar with both the test and the disease and be experienced at testing behaviourally challenging children.

Selection of appropriate cognitive assessments presents a further difficulty. Individuals may span a range of abilities and ages, from normal to severely impaired, and from infancy to adulthood. A single instrument for cognitive assessment is therefore unlikely to be sufficient [67]. In clinical trials, this variability may be partly mitigated by selection and stratification of subjects to create a more homogeneous patient population. An appropriately sensitive measure can then be selected.

The scale should be appropriate for tracking longitudinal development. Test batteries should be short and focused to avoid fatigue, should not rely on verbal-based subtests and should be supplemented by parent reported measures. The difficulty level should be appropriate for the disease and up to date normative data should be available. In addition, the test should be available and familiar to researchers internationally and translated into several languages [65, 67], as multi-centre studies are a necessity in this rare disease. Based on these criteria, we advocate the use of the Bayley Scales of Infant and Toddler Development, Third Edition (BSID-III) in children with an expected developmental age up to 42 months, or the Kaufman Assessment Battery for Children, Second Edition (KABC-II).

Finally, there are challenges in how data should be presented and interpreted. Historically, standard scores have been preferred to age equivalent scores (AgeEqSs) or developmental quotient (DQ) as the latter do not take into account the range in normality, and present challenges in statistical analysis as the intervals are unequal (the same change in raw score may represent quite different changes in AgeEqSs or DQ at different ages) [65, 67]. However, standard scores may be less useful in this patient group. Given the nonlinear trajectory of development in MPS III, treatment effects on cognitive outcomes need to be assessed based on a developmental growth curve which cannot be calculated from standard scores. Standard scores also have a floor below which low-functioning children may fall, thereby reducing the sensitivity of the test for tracking longitudinal development [67]. We therefore advocate the use of AgeEqSs to assess the relative rate of development in MPS III. However, AgeEqSs are more difficult to manage statistically and DQ may allow for easier comparison between subjects.

Assessment of adaptive behaviour using the Vineland Adaptive Behaviour Scale, Second Edition (VABS-II) may be a useful additional measure of development. Due to the multiple challenges of assessment in this group, standardised cognitive assessments cannot always be administered and therefore, despite not being a true neurocognitive test, the VABS-II at least provides an account of development from parent report which can be corroborated from observation of the child in clinic.

5. Behavioural manifestations are hugely relevant to quality of life and the impact on families and should therefore be considered in the development of clinical trials. Potentially useful measures of behavioural manifestations include the Sanfilippo Behaviour Rating Scale and actigraphy.

Behavioural manifestations include hyperactivity, aggression, temper tantrums, unusual affect and orality [70]. Sleep disturbance may be considered part of the behavioural phenotype and correlates with behavioural disturbance during the daytime [70, 71]. Elements of the behavioural phenotype overlap with features of autism spectrum disorders, in particular impaired social communication, reported in both MPS IIIA and IIIB [72, 73] and in some cases this has led to the misdiagnosis of MPS III as autism [26]. Behavioural manifestations have a huge impact on families of children with MPS III, who often consider these to be more challenging than physical symptoms [66].

Behavioural manifestations appear at a specific point in the disease course and certain behaviours such as hyperactivity and aggression may decline with disease progression. Behavioural disturbance may also be less apparent in attenuated patients. Robust natural history data on behavioural outcome measures is therefore required for these to be used as endpoints, and not enough data currently exists for this to be recommended as a primary endpoint.

The Sanfilippo Behaviour Rating Scale (SBRS) has been developed as a disease specific measure designed to track behavioural changes through different stages of disease, and to encompass the full range of behaviours seen in MPS III [74]. These include orality and dampened emotional expression, including lack of fear, which appear to correlate with structural brain changes, specifically amygdala volume [74, 75]. Further experience and validation of SBRS in Sanfilippo patients would be beneficial.

Actigraphy measures level of activity over time and allows discrimination between states of sleep and wakefulness [76]. The circadian rhythm in MPS III appears to be fragmented, with a phase delayed sleep-wake cycle in some children [77]. Children have higher levels of activity in the early morning and abnormalities in endogenous melatonin production [76–78]. Though the relationship between actigraphic data and daytime behaviour needs to be further examined, actigraphy allows measurement of sleep and circadian rhythm functioning, and may be a useful additional measure in clinical trials.

6. MPS III has a huge impact on the life of a child and on the broader family and community. Standardised quality of life measures currently available are likely to be inadequate. Contributing factors to quality of life may change, such as the presence or absence of hyperactivity and sleep disturbance. What constitutes good quality of life is therefore likely to vary during the disease process. Quality of life measures for MPS III that take these factors into account need to be developed.

MPS III has a significant impact on the lives of a child and their family. In addition to the symptoms already described, chronic pain is increasingly recognised as a feature [79]. Families have described having a child with MPS III as ‘devastating,’ with significant emotional and financial impact, and stresses on marital, social and family relationships [80]. Sleep disturbance, agitation, repetitive behaviours and diarrhoea are reported to be the most frequent and challenging symptoms for families to deal with, and behavioural symptoms can be relentless, resulting in anger, frustration and mental and physical exhaustion [66]. Though the impact of behavioural symptoms may decrease as the disease progresses, intellectual and physical ability continues to deteriorate and may affect parental psychological functioning [81]. Parents of children with MPS III may also be at risk for problems with anxiety and depression [81].

Currently available, non-disease specific, standardized quality of life measures (QoL) are likely to be inadequate given the changing nature of factors contributing to quality of life during the disease process, and have so far proved inadequate in other MPS trials. Scales such as SBRS and the MPS III disability scale [82] are not fully validated and may not reflect some aspects of QoL. Activities of daily living, though not a QoL measure, may have functional relevance (independent eating, ambulation etc.) and therefore measures of adaptive behaviour such as VABS-II may be useful surrogates, as they include socialization and daily living measures. In one study, assessments of parental anxiety and depression using the Beck Depression Inventory (BDI) were used as surrogate markers of the effects of risperidone on behaviour [83] in an ongoing clinical trial of genistein in MPS III such measures enabled the identification of parents with clinical levels of psychological distress (Stewart Rust, personal communication). Assessment of parental mood using the BDI-II may therefore be useful.

QoL measures are of interest to authorities making funding decisions regarding potential MPS III therapies and there is therefore a need for disease-specific QoL measures to be developed or to select QoL measures sensitive to MPS III. Such measures need to directly assess the dimensions that parents or caretakers feel are important. While these measures will likely not be sufficiently sensitive as primary endpoints, it is critical that parents feel the applied treatment have a meaningful and measureable impact for the patient and family. Developing such a measure requires considerable resources. A joint effort that includes academic, clinical, patient groups and industry should be considered. Certain research groups are also developing qualitative research, using parent interviewing, to capture meaningful aspects of the disease and how it affects patient and family life (Samantha Parker, personal communication).

7. Measures of CSF heparan sulfate are generally agreed to be the best current biomarker for neurological disease, though there is continuing debate regarding the best methods of measurement of heparan sulfate and heparan sulfate derived structures. While it is possible to measure heparan sulfate in blood and urine, these have less relevance to neurological outcomes in clinical trials.

Due to the slowly progressive nature of MPS III, small numbers of patients, clinical heterogeneity, and the difficulties of quantifying neuropsychological outcomes, biomarkers are critical to the development of new therapies. An ideal biomarkers is easily and reliably measurable, correlates closely with disease burden and relevant clinico-pathological parameters, and responds rapidly to treatment [84]. In MPS III there is a need for biomarkers to reflect CNS disease progression in particular. Biomarkers may be the best available measure of short-term treatment effects, particularly during the short time period of a clinical trial or in early treated patients, and given the difficulties associated with cognitive outcome measures. Urinary glycosaminoglycans (uGAG), while commonly used in MPS disorders, may differentially reflect GAG burdens in different organs, and, due to the blood-brain barrier, may not effectively reflect burden of disease in the brain. Measurement of cerebrospinal fluid (CSF) HS is a logical option to consider as HS is the primary storage metabolite in MPS III. CSF HS has been shown to respond to treatments in MPS I [85]. There is ongoing debate about which of the multiple methods of quantitating HS and HS-derived structures [86–89] may be the most suitable. Obtaining CSF samples is invasive, requiring lumbar puncture, but CSF HS is more likely to reflect neurological disease than urine or plasma HS. However, although CSF HS may reflect substrate concentrations in intrathecal spaces, it will still be only a surrogate measurement of substrate storage in brain tissue. Recent work in large animal models suggests that CSF HS reduction after intra-CSF enzyme treatment was greater in brain cortex than in deeper brain structures [90]. Its use as a biomarker may therefore be of more relevance for interventions not administered to the CSF.

Surrogate GAG biomarkers that are directly dependent on HS levels are also possibilities. For example, heparin cofactor II thrombin complex [91] has some value in responses to treatment in MPS I, but is more dependent on dermatan sulfate for complex formation than HS [92] and although it can be used to distinguish MPS III patients [93], it will likely be more limited than direct measurements of HS.

A recently published natural history study suggests that CSF enzyme activity in untreated MPS III patients is lower than in controls, and this appears to be more discriminatory for MPS IIIB than for MPS IIIA [57]. Changes in CSF enzyme activity may be a relevant marker in certain trials, such as gene therapy trials where it may reflect the degree of expression of therapeutic protein in the brain [94], or systemically delivered ERT where it may reflect the degree to which enzyme crosses the blood-brain barrier. However, it is less likely to be useful for interventions such as intrathecal ERTs or SRTs. Ultimately, however, CSF enzyme activity does not reflect the disease modifying effect of a therapy and CSF HS may be more meaningful in this regard.

8. Further characterisation of the associations between biomarkers and clinical outcomes in MPS III should be a goal of both natural history studies and long-term follow up of interventional trials, preferably in prospective studies.

Though many putative biomarkers for MPS III exist, uncertainties remain as to how accurately they correspond to disease outcomes and response to therapy. Detailed characterization of associations with clinical outcomes will be required for both existing and novel biomarkers. This has been demonstrated effectively in retrospective studies in MPS I with relationships established between uGAG and plasma enzyme level after treatment [95], urine and plasma HS against uGAG after treatment [89] and urine and plasma HS against both enzyme and clinical outcomes such as sleep disordered breathing [96].

9. Neuroimaging, such as measurement of cortical volume by MRI, is associated with deterioration in neurocognitive outcomes but is more difficult in younger patients and may at best be a useful secondary endpoint.

A range of MRI brain abnormalities have been described, including cortical atrophy and increased ventricular volume [97]. Quantitative methods such as volumetric analyses may be functionally relevant. Reduction of amygdala volume and hippocampal volume has been reported to be associated with lack of fear and social/emotional dysfunction respectively [74]. Cortical grey matter volume loss is associated with a decline in DQ [58] and may be an appropriate marker of disease-related brain changes, and a useful secondary endpoint in trials. This analysis can be automated but must be supplemented with manual editing due to inaccuracies due to lack of clear gray-white differentiation. As gray matter volumes cannot be accurately analyzed for children under 2 years of age, ventricular volume, which tends to increase over time [58], may be a useful measure.

10. Given the progressive nature of MPS III, lack of effective therapies and small numbers of patients, there are significant challenges to conducting interventional trials with formal comparator groups. A detailed understanding of the natural history in this disease is therefore essential.

Conducting interventional clinical trials with a formal comparator group presents challenges in MPS III. Given the lack of effective therapies, families may be anxious about being allocated to a non-interventional arm of a study. One method of overcoming this is to use a partial randomized crossover design, either by including an open label treatment phase in the study or allowing participants who completed a primary study with a comparator group to enroll in an open label long-term extension study [56, 98]. However, disease progression during the initial part of the study may remain a source of anxiety for families. Where interventions are invasive (e.g. brain injections, intrathecal therapies requiring access device implantation), a formal ‘placebo’ approach may not be appropriate. This can be partly mitigated by using a ‘no treatment’ comparator group where only the assessors of the primary endpoint are blinded, as were the testers for neuropsychological assessments in a randomized controlled trial of intrathecal ERT in MPS II [98]. However, clinical variability and small numbers of patients limits the utility of comparator groups. A more detailed understanding of the natural history of individual subtypes of MPS III, together with consensus on what outcomes should be measured, may enable future interventional trials to be conducted without the need for formal comparator groups, as has previously been possible with other lysosomal disorders [99, 100].

11. Due to the numerous challenges in performing research in MPS III and the urgent need for the development of effective therapies, all studies in this area should be published as soon as possible, and within a year of study completion. This includes both interventional trials and natural history studies, especially those involving invasive investigations. For natural history studies, an ideal situation would involve post-publication sharing of raw data in a central independent repository.

Good quality natural history data is vital to the development of clinical trial designs and analysis. Given the multiplicity of potential treatment approaches, the conduct of individual natural history studies by multiple groups both delays the evaluation of therapeutic interventions and reduplicates efforts, both of which will have a considerable impact on families deciding on the best course of action for their child. In addition, conducting natural history studies or interventional studies with placebo or no treatment comparator groups may no longer be possible once the first effective treatments become available. Cohorts of good quality published data are therefore required now. Natural history data should be published in a timely manner after completion and ideally in a central repository – preferably held in a “neutral” academic setting or by patient organisations. Interventional trials should similarly be published within 1 year of study completion. Industry and academic groups must collaborate to facilitate these goals.

## Discussion

**Fig. 1:**
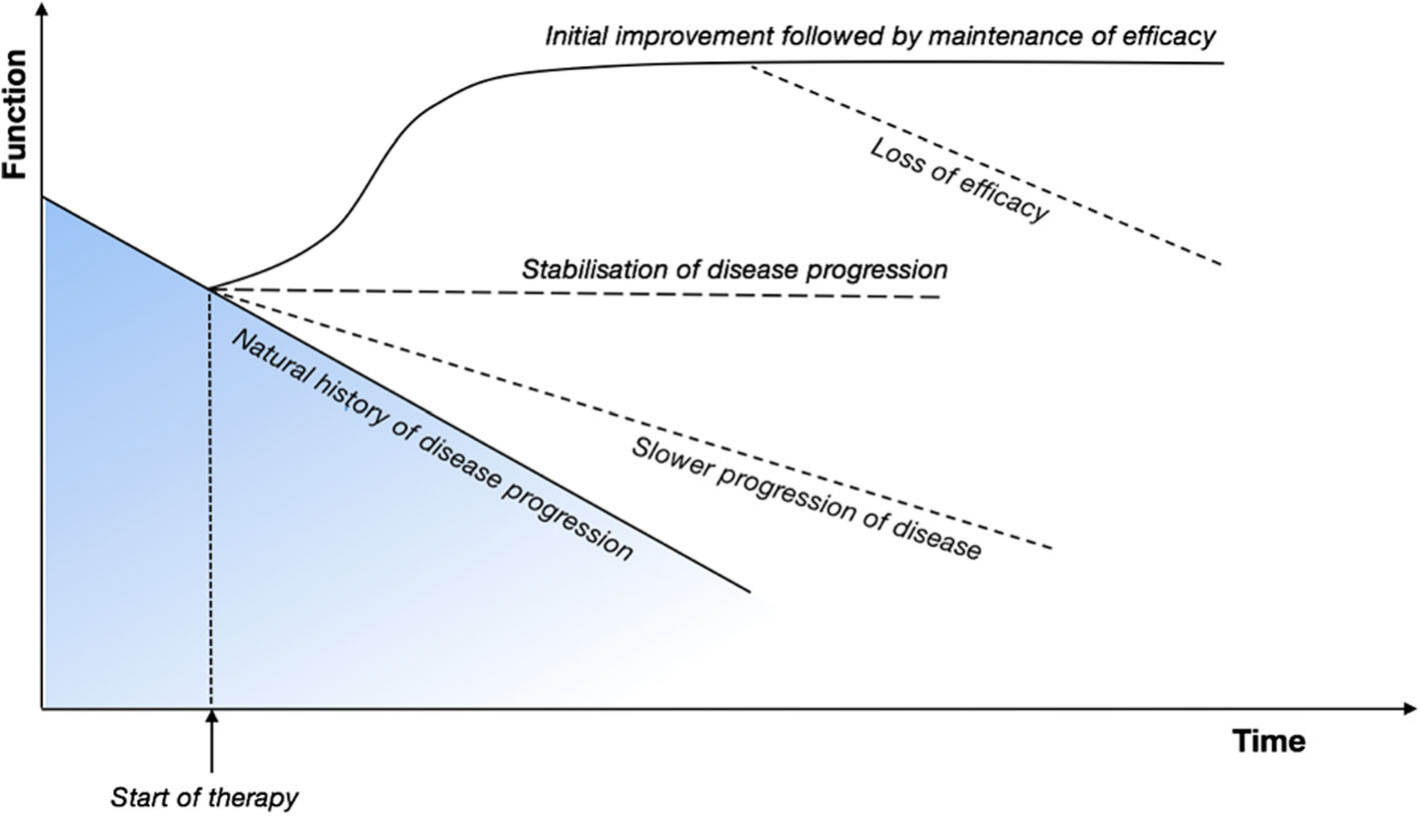
Possible responses to therapy in a progressive disorder such as MPS III. (Courtesy of Ed Wraith)

There are considerable challenges to conducting clinical research in MPS III. In a progressive neurodegenerative condition, it is far from clear what the criteria for an effective therapy should be: reversal of decline, a steady state, or slowing of progressive decline? (Fig. 1). It is unlikely that any one marker of disease will be sufficient to evaluate this. Comparisons to MPS I may be relevant, as this also involves HS storage and neurological decline in severely affected individuals, but there is an effective CNS treatment (HSCT). HSCT can prevent further neurological decline in MPS I, and children continue to develop skills, albeit at a slower rate. This could therefore be considered the goal of an ideal therapy for MPS III. In MPS I, it is evident that early treatment minimises residual disease burden and improves both neurological and somatic long-term outcomes [101]. Given the likelihood that neurological disease is similarly irreversible in MPS III, early treatment is to be recommended, and this would be consistent with observations in mouse models [102]. As MPS III is often not diagnosed before the onset of neurological decline, the establishment and implementation of a newborn screening programme may be the only feasible way to achieve this. The ability to identify children with MPS III early would enable better opportunities for informed disease management and clinical trials participation.

Due to the slowly progressive nature of the disease, assessing the clinical outcomes of interventions within the relatively short time frame of clinical trials can be difficult. Long-term follow-up studies are valuable but given the numerous potential treatment strategies in development and the lack of effective therapy, there is a need for development of short-term predictors of longer-term outcomes, including the detailed characterisation of the association between disease biomarkers and clinical outcomes.

The wide clinical heterogeneity of the condition, combined with the relatively small numbers of patients presents difficulties in interpretation and statistical analysis of data. One approach to mitigating this in clinical trials may be through subject selection and stratification to create a more homogenous patient population. Ideally MPS IIIA, B, C and D should be considered separately, though in one ongoing trial where this was not possible, MPS IIIC patients were stratified separately (due to known slower progression of disease in MPS IIIC) [56]. Individuals likely to have rapidly progressive disease should be considered together, and this may be more feasible in MPS IIIA where specific mutations associated with rapid progression are recognised. However, a more detailed understanding of genotype-phenotype correlations is required with respect to other MPS III subtypes and slowly progressive phenotypes.

Evaluating and quantifying clinical outcomes in MPS III can be difficult, particularly neuropsychological assessments, and this is compounded by clinical heterogeneity and small numbers of patients. There is therefore a need for consensus over which outcome measures and instruments should be used in clinical trials. We have outlined certain broad recommendations here but this could be further developed by the construction of a ‘core outcome set’ for MPS III, i.e. an agreed minimum set of outcomes that should be measured and reported in all clinical trials [103]. This standardised approach potentially allows for robust meta-analysis in future clinical research which will be of considerable value in such a rare disease.

Conducting effective clinical research in MPS III requires a body of robust and quantitative natural history data. Though the natural history of MPS III has been well described in a narrative sense, it is only recently that good quality, prospectively collected longitudinal data has been published. Timely publication of natural history studies, sharing of data and a consistency of approach to outcome measures, potentially allowing for meta-analysis, will be essential to improve the quality of research in this field.

## Conclusions

There is a pressing need to move forward with clinical research in a disease with huge impact on quality of life for affected children and families for which there remains no effective therapy. To do so most effectively will require an open, systematic and collaborative approach from academic groups, clinicians, patient groups and industry. As part of this process, we have made a series of recommendations on the conduct of clinical research and selection of outcome measures, and we emphasize the importance of timely publication and sharing of natural history data.

## Abbreviations

AgeEqSs: Age equivalent scores
BSID-III: Bayley Scales of Infant and Toddler Development, Third Edition
CNS: Central nervous system
CSF: Cerebrospinal fluid
DQ: Developmental quotient
ENT: Ear, nose and throat
ERT: Enzyme replacement therapy
GAG: Glycosaminoglycan
GNS: N-acetylglucosamine-6-sulfatase
HGSNAT: Heparan-α-glucosaminide N-acetyltransferase
HS: Heparan sulfate
HSCT: Haematopoietic stem cell transplantation
KABC-II: Kaufman Assessment Battery for Children, Second Edition
MPS I: Mucopolysaccharidosis Type I
MPS III: Mucopolysaccharidosis Type III
NAGLU: N-acetyl-α-glucosaminidase
QoL: Quality of life
SBRS: Sanfilippo Behaviour Rating Scale
SGSH: N-sulfoglucosamine sulfohydrolase
SRT: Substrate reduction therapy
uGAG: Urinary glycosaminoglycans
VABS-II: Vineland Adaptive Behaviour Scale, Second Edition
